# Right-to-left shunt-associated brain functional changes in migraine: evidences from a resting-state FMRI study

**DOI:** 10.3389/fnhum.2024.1432525

**Published:** 2024-08-30

**Authors:** Wenfei Cao, Lei Jiao, Huizhong Zhou, Jiaqi Zhong, Nizhuan Wang, Jiajun Yang

**Affiliations:** ^1^Department of Neurology, Shanghai Sixth People's Hospital Affiliated to Shanghai Jiao Tong University School of Medicine, Shanghai, China; ^2^Department of Chinese and Bilingual Studies, The Hong Kong Polytechnic University, Kowloon, Hong Kong SAR, China; ^3^School of Biomedical Engineering, ShanghaiTech University, Shanghai, China

**Keywords:** migraine, right to left shunt, functional magnetic resonance imaging, amplitude of low-frequency fluctuation, functional connectivity, brain network

## Abstract

**Background:**

Migraine, a neurological condition perpetually under investigation, remains shrouded in mystery regarding its underlying causes. While a potential link to Right-to-Left Shunt (RLS) has been postulated, the exact nature of this association remains elusive, necessitating further exploration.

**Methods:**

The amplitude of low-frequency fluctuation (ALFF), fractional ALFF (fALFF), regional homogeneity (ReHo) and functional connectivity (FC) were employed to investigate functional segregation and functional integration across distinct brain regions. Graph theory-based network analysis was utilized to assess functional networks in migraine patients with RLS. Pearson correlation analysis further explored the relationship between RLS severity and various functional metrics.

**Results:**

Compared with migraine patients without RLS, patients with RLS exhibited a significant increase in the ALFF within left middle occipital and superior occipital gyrus; In migraine patients with RLS, significantly reduced brain functional connectivity was found, including the connectivity between default mode network and visual network, ventral attention network, as well as the intra-functional connectivity of somatomotor network and its connection with the limbic network, and also the connectivity between the left rolandic operculum and the right middle cingulate gyrus. Notably, a significantly enhanced functional connectivity between the frontoparietal network and the ventral attention network was found in migraine with RLS; Patients with RLS displayed higher values of the normalized clustering coefficient and greater betweenness centrality in specific regions, including the left precuneus, right insula, and right inferior temporal gyrus. Additionally, these patients displayed a diminished nodal degree in the occipital lobe and reduced nodal efficiency within the fusiform gyrus; Further, the study found positive correlations between ALFF in the temporal lobes, thalamus, left middle occipital, and superior occipital gyrus and RLS severity. Conversely, negative correlations emerged between ALFF in the right inferior frontal gyrus, middle frontal gyrus, and insula and RLS grading. Finally, the study identified a positive correlation between angular gyrus betweenness centrality and RLS severity.

**Conclusion:**

RLS-associated brain functional alterations in migraine consisted of local brain regions, connectivity, and networks involved in pain conduction and regulation did exist in migraine with RLS.

## 1 Introduction

In the context of International Classification of Headache Disorders, 3rd edition (ICHD-3) (Arnold, 2018), migraine is characterized by specific attributes, including unilateral localization, moderate to severe intensity, recurrent episodes, pulsatile headache, and a duration lasting for 4–72 h. Migraine, a prevalent and incapacitating condition, exerts detrimental impacts across multiple facets of individuals’ lives, encompassing marital bonds, parental duties, emotional stability, and more (Buse et al., 2019). This intricate disorder can be induced by a variety of factors, including physical exertion, sleep irregularities, dietary choices, meteorological variations, emotional fluctuations, among others (Chądzyński et al., 2019; Moon et al., 2017; Fernández-de-Las-Peñas et al., 2017). Nonetheless, the comprehensive pathophysiological underpinnings of migraine remain partially elucidated. An increasing body of research suggests the involvement of various mechanisms, encompassing cortical spreading depression (CSD), vascular alterations, neurogenic inflammation, release of vasoactive substance, disturbances in energy metabolism and genetic factors (Domitrz and Cegielska, 2022; Goadsby and Holland, 2019; Stanyer et al., 2021). In light of this multifaceted backdrop, factors exhibiting correlations with migraine comprise sleep disorders (Holland et al., 2018; Kim et al., 2019), dysbiosis of gut microbiota (Wen et al., 2019; Arzani et al., 2020), levels of sex hormone (Warnock et al., 2017), and genetics (Hautakangas et al., 2022; Sutherland et al., 2019). Furthermore, in recent decades, the association between RLS and migraine has been the subject of debate (Zhao et al., 2021; Takagi et al., 2016).

RLS denotes an anomalous shunting pathway connecting the venous and arterial circulations, with the most prevalent etiology being the patent foramen ovale (PFO), accounting for 95% (Liu K. et al., 2020; Liu L. et al., 2020). The contrast-enhanced transcranial doppler (cTCD) is routinely employed in the clinical assessment of individuals suspected of RLS, renowned for its exceptional sensitivity in detecting the condition (Zhang et al., 2021). The association between RLS and migraine stems from the pioneering work of Del et al., who initially disclosed a heightened prevalence of RLS among patients with migraine with aura when compared to healthy individuals (Del Sette et al., 1998). Subsequent observational studies have consistently supported this association (Lip and Lip, 2014; Tian et al., 2019; Zhao et al., 2021; Tang et al., 2022). Due to variations in prevalence, a plausible association between RLS and migraine has been suggested. The hypothesis posits that RLS may facilitate the passage of vasoactive substances such as 5-HT, nitric oxide, and kinin directly into the cerebral circulation, potentially triggering migraine attacks (Dalla Volta et al., 2005; Wilmshurst and Nightingale, 2006). Moreover, the presence of RLS is implicated in the formation of microemboli, initiating cortical spreading depression (CSD), which, in turn, may activate the trigeminal neurovascular system, leading to headaches (Ashina et al., 2019; Sevgi et al., 2012).

Our hypothesis posits the existence of differences in certain brain structures and functions between migraine patients with RLS and those without it. Notably, recent years have witnessed a growing body of neuroimaging evidence revealing pronounced disparities in white matter hyperintensities (WMH) among migraine patients, particularly when comparing those with and without RLS (Cao et al., 2022). Specifically, these differences manifest in significant variations in the prevalence, location, and volume of WMH between migraineurs with and without RLS (Park et al., 2011; Yoon et al., 2012; Iwasaki et al., 2017). Resting-state functional magnetic resonance imaging (rs-fMRI) is a widely used method for detecting the dynamics of brain function in patients under resting conditions (Vakamudi et al., 2012). The analysis of rs-fMRI data encompasses multiple approaches (Zang et al., 2007; Wang et al., 2013; Wang et al., 2012; Tang et al., 2017; Wang et al., 2014), broadly classified into functional segregation and functional integration methods (Lv et al., 2018). Amplitude of Low-Frequency Fluctuations (ALFF), fractional ALFF (fALFF) and Regional Homogeneity (ReHo) primarily focus on evaluating local functional characteristics of specific brain regions (Zou et al., 2008; Yu et al., 2021; Zang et al., 2004). In contrast, functional integration, represented by methods such as functional connectivity (FC) and graph theory analysis, delves into the assessment of inter-regional functional interactions, essentially exploring the overall organization of the brain as an integrated network (Wang et al., 2017; Wang et al., 2015; Tang et al., 2015; Tang et al., 2017; Wang et al., 2018; Shi et al., 2021; Yan et al., 2022; Sporns, 2018; Bullmore and Sporns, 2009). Numerous rs-fMRI studies have been conducted to elucidate the physiological underpinnings of migraine (Messina et al., 2022). In particular, aberrant descending pain modulatory pathway prior to the migraine attack, abnormal thalamo-cortical and frontoparietal pathways involved in pain transmission and modulation have been observed in migraine individuals (Nie et al., 2021; Lim et al., 2021; Mungoven et al., 2022).

In this study, we posit a potential correlation between RLS and functional alterations in integration and segregation in individuals with migraines. To examine this hypothesis, the study meticulously explores RLS-associated brain functional aberrations in migraine individuals with and without RLS The overarching goal is to unravel the underlying connection between RLS and migraines, contributing to a comprehensive understanding of this intricate interplay.

## 2 Materials and methods

### 2.1 Participants

The cohort of migraine patients, seeking medical attention from 2020 to 2022, was meticulously assembled from the Department of Neurology at the Shanghai Sixth People’s Hospital, which is affiliated with the Shanghai Jiao Tong University School of Medicine. All participants provided their explicit informed consent by signing the requisite agreement, signifying their willingness to participate in this study. Moreover, it is important to emphasize that this study received the formal approval of the Ethics Committee at the Shanghai Sixth People’s Hospital, Shanghai Jiao Tong University School of Medicine.

In adherence to the inclusion criteria for migraine patients, the following prerequisites were established: (1) Conformance to the diagnostic criteria for migraine as stipulated in ICHD-3; (2) Age range of 14–70 years; (3) Inclusion in the study was contingent upon subjects being within the interval between migraine attacks.

Conversely, a series of stringent exclusion criteria were meticulously applied, including: (1) The presence of other primary and secondary headache disorders; (2) Concurrent manifestation of mental illnesses; hypertension, vascular/heart disease, and any major systemic disorders; (3) A history of substance addiction involving alcohol or drugs; (4) The presence of contraindications that rendered individuals unsuitable for MRI or cTCD examinations.

All the participants provided written informed consent to participate in the current study. The study was registered with Clinical Trial (ChiCTR2300067636) and obtained ethical approval from the Ethics Committee of Shanghai Sixth People’s Hospital Affiliated to Shanghai Jiao Tong University School of Medicine (Approval No. 2022-KY-194(K)).

### 2.2 Data acquisition

#### 2.2.1 Demography and clinical data

Demographic data, comprising age, gender, body mass index (BMI), and educational history were meticulously acquired. Furthermore, a structured diagnostic interview was conducted to elicit comprehensive information concerning the patients’ symptoms, including the presence or absence of migraine triggers, aura phenomena, the frequency (the mean number of migraine attacks per month) and duration (the mean duration of each migraine attack) of migraine episodes, remission and exacerbation factors, and any concomitant symptoms experienced. Notably, a Visual Analog Scale (VAS) was utilized as a quantitative measure to assess the severity of pain, with progressively decreasing scores indicating lesser pain intensity, and conversely, escalating scores denoting increasingly severe pain.

#### 2.2.2 Contrast-enhanced transcranial Doppler

All participants underwent a cTCD examination, administered by skilled physicians to ascertain the presence of RLS. During this examination, the patients reclined in a supine position, and a three-way catheter was inserted into the left elbow vein to establish a venous access. Two 20 mL syringes were connected to the catheter, one containing a solution comprising 8 mL of physiological saline, 1 mL of air, and 1 mL of autologous blood. The contents of the two syringes were then rapidly interchanged to generate an air microbubble suspension. Subsequently, this microbubble suspension was promptly injected into the elbow vein. Simultaneously, ultrasonic monitoring of microembolic signals in the middle cerebral artery (MCA) was conducted using a transcranial Doppler ultrasound probe via the temporal bone window, with recordings lasting for 20 s. This examination was performed both at rest and during the Valsalva maneuver. The RLS was graded based on the maximum number of microemboli observed. Specifically, if 1–10 microbubbles (one side) were detected, it was categorized as a small RLS; if 11–20 microbubbles (one side) were observed, it was classified as a medium RLS. Moreover, if more than 20 microbubbles (one side) or curtain patterns were noted, it was designated as a large RLS (Zhang et al., 2021).

#### 2.2.3 FMRI data acquisition

Initially, each subject underwent a comprehensive brain MRI examination encompassing various sequences, such as T1-weighted images (T1WI), T2-weighted images (T2WI), fluid attenuated inversion recovery (FLAIR) images, and diffusion-weighted imaging (DWI) images, with the final diagnosis being conferred by a qualified medical specialist. Any data presenting secondary intracranial lesions were subsequently excluded from the analysis, while fMRI data acquisition proceeded for those without such lesions. All MRI and fMRI scans were executed employing a 3.0 T GE MRI scanner (SIGNA, GE Healthcare). Furthermore, participants were instructed to remain in an awakened state with their head securely immobilized, maintaining physical relaxation, and refraining from intentional cognitive activity throughout an 8-min resting-state fMRI session.

The resting-state fMRI parameters of all participants in this study adhered to the following standards: repetition time (TR) =3,000 ms, echo time (TE) =30 ms, field of view (FOV) =240 mm × 240 mm, matrix =128 × 128, slice thickness = 4 mm, flip angle =90°, comprising 38 axial slices arranged in parallel, with 160 time points acquired.

### 2.3 Data preprocessing

**Figure 1:**
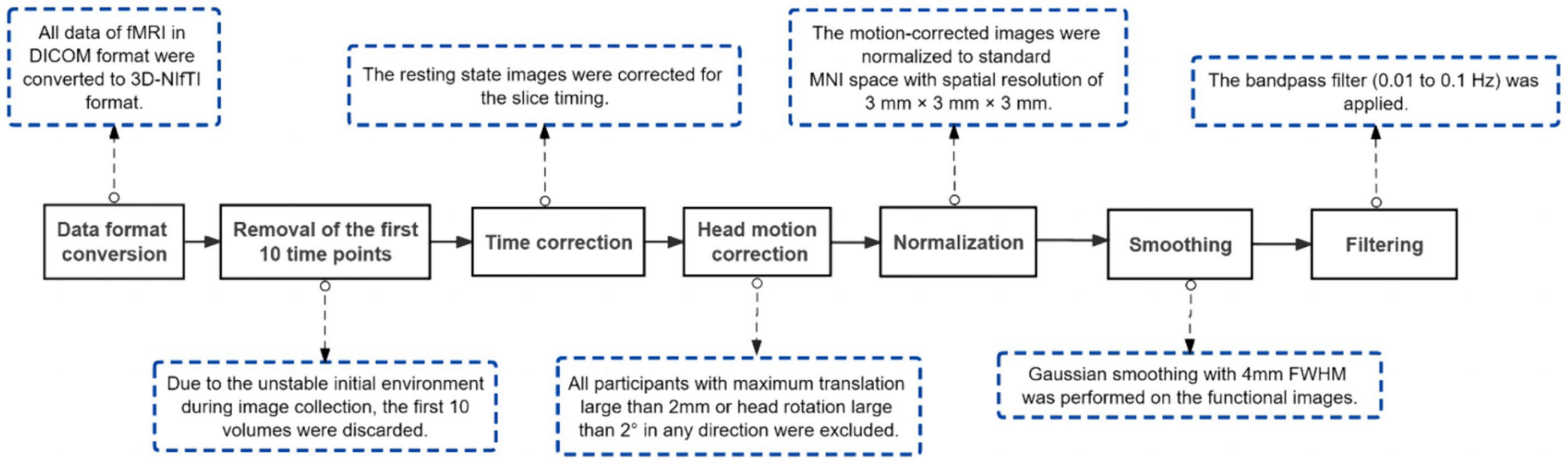
Procedures of pre-processing. DICOM, Digital Imaging and Communications in Medicine; NIfTI, Neuroimaging Informatics Technology Initiative; MNI, Montreal Neurological Institute; FWHM, Full Width Half Maximum.

Preceding data analysis, a sequence of preprocessing procedures and stringent quality control measures were meticulously executed on rs-fMRI data. These tasks were diligently carried out using the Data Processing and Analysis of Brain Imaging (DPABI) toolbox (version 6.1^1^) based on MATLAB (The Math Works, Natick, MA, United States) software, to remove the effects of data acquisition, physiological noise, and individualized variations in the subject’s brain, as well as to ensure the confidence and sensitivity of the group-level analysis (Yan et al., 2016). Figure 1 specifies the detailed steps of preprocessing and its role.

### 2.4 ALFF analysis and ReHo analysis

Preceding the application of filtering, the ALFF and fALFF was computed using the DPABI software (Yan et al., 2016; Yan and Zang, 2010), relying on the preprocessed dataset. This entailed the transformation of the time series data of an individual voxel into a frequency spectrum by means of the Fourier transform. Subsequently, the summation of amplitudes within the frequency range of 0.01–0.1 Hz was executed to derive the ALFF and fALFF (Lv et al., 2018; Zuo et al., 2010; Biswal et al., 1995). This procedure was systematically applied to all voxels throughout the entire brain, thereby producing the comprehensive ALFF map representing the entire cerebral structure of the subject.

The ReHo maps for the subjects were created by computing the Kendall’s Coefficient of Concordance between the temporal series of each voxel and its adjacent 26 voxels, operating at a voxel-by-voxel level (Zang et al., 2004). Utilizing the DPABI software, the ReHo analysis was executed on the preprocessed data prior to smoothing. Following this, spatial smoothing was applied to the obtained ReHo maps, aiming to amplify the signal-to-noise ratio and rectify registration inaccuracies stemming from the normalization procedure.

### 2.5 Network construction and analysis

Brain network construction and subsequent analysis were executed using the DPABI NET (version 1.1) toolbox. The anatomical automatic labeling (AAL) template (available at: http://www.gin.cnrs.fr/en/tools/aal/), a widely recognized brain atlas, was adopted to partition the entire brain into a total of 116 distinct brain regions (Tzourio-Mazoyer et al., 2002). It is worth noting that 26 of these regions are located within the cerebellum. Consequently, only the 90 regions of interest (ROIs) within the cerebral cortex were chosen as nodes for network construction. The average time series of each ROI were extracted, and the Pearson’s correlation coefficient between the average time series of each two ROIs was calculated, thus obtaining a 90 × 90 two-dimensional matrix to establish the brain functional network. Similarly, we have constructed brain functional networks based on the Dosenbach’s 160 atlas (Dosenbach et al., 2010), and furthermore, we have conducted a comprehensive mapping analysis of these networks utilizing the Yeo2013 template (Yeo et al., 2011). Additionally, a range of sparsity settings, varying from 0.01 to 0.5, were applied to construct a brain-weighted network. This range ensured that a connection matrix involving all 90 nodes was encompassed within this specified sparsity range.

Complex-network (graph) analysis was performed using Brain Connectivity Toolbox^2^ (Rubinov and Sporns, 2010) while the BrainNet viewer^3^ was utilized to display results (Xia et al., 2013). Brain network topology analysis includes “small world” properties as well as node properties. In line with the prior literature (Yan et al., 2013), we computed each small-world property’s value across 50 different sparsity levels. Furthermore, we determined the area under the curve (AUC) for each small-world metric within the sparsity range of 0.01–0.34, allowing the more sensitive alteration detection in small-world network of brain. Subsequently, we derived the values for the following small-world properties at each sparsity level: characteristic shortest path length (L_p_), clustering coefficient (C_p_), normalized clustering coefficient (γ), normalized characteristic shortest path length (λ), small-worldness (σ), local efficiency (E_loc_), global efficiency (E_glob_), degree centrality, betweenness centrality, and nodal efficiency (Yang et al., 2021).

### 2.6 Statistical analysis

Demographic characteristics, including age, gender, and years of education, and clinical features such as migraine duration, frequency, and VAS score, underwent rigorous statistical analysis utilizing Statistical Product and Service Solutions (SPSS) software.^4^ Categorical variables were assessed with chi-square tests, and numerical variables were statistically evaluated through independent samples *t*-tests and non-parametric tests. A significance level of *p* < 0.05 indicated statistical significance.

Two-sample *t*-tests were conducted for ALFF, fALFF and ReHo in both groups, utilizing age and gender as covariates. In order to minimize the false-positive rates, a stringent correction for multiple comparison was applied at the clump level (voxel *p* < 0.001, cluster *p*-value < 0.05, corrected using GFR) in this study. The present study reveals the surviving corrected clumps. ALFF, fALFF and ReHo values were extracted from the migraine with RLS group for subsequent Pearson’s linear correlation analysis with RLS grading. The statistical threshold was established at *p* < 0.05, with a requirement of a cluster size exceeding 60.

Two-sample *t*-tests were carried out to assess functional connectivity differences between the two groups, with a significance threshold set at *p* < 0.001. Furthermore, small-world properties (with a significance threshold at *p* < 0.05) and nodal properties (with a significance threshold at *p* < 0.01) were subjected to two sample *t*-tests, while accounting for age and gender as covariates. The surviving nodes were then reported. In all Pearson’s linear correlation analyses in the current study involving RLS grading, the significance level was set at *p* < 0.05.

## 3 Results

### 3.1 General characteristics and clinical features

The clinical and demographic data of all patients are presented in Table 1. No significant differences were observed between the two groups concerning gender, age, years of education, duration of migraine (years), frequency (in times per month) and VAS scores. In total, 32 patients were enrolled in this study, comprising 14 migraine patients with RLS and 18 migraine patients without RLS. Within the RLS group, five patients with large RLS, three had moderate RLS, and six showed mild RLS.

**Table 1:** Characteristics and clinical profiles of migraine patients with and without RLS.

Characteristics	Migraine		*p* value
	With RLS (*n* = 14)	Without RLS (*n* = 18)	
**Sex, *n*%**			
Male	3 (21.43%)	1 (5.56%)	
Female	11 (78.57)	17 (94.44%)	
Age (years)	36.71 ± 6.62	41.56 ± 12.41	0.168
Years of education	13.71 ± 2.37	13.11 ± 4.01	0.889
Migraine duration (years)	12.71 ± 9.45	16.5 ± 12.49	0.401
Frequency (times/month)	3.69 ± 3.24	1.92 ± 1.46	0.086
VAS	8.11 ± 0.92	7.83 ± 1.83	0.969

RLS, Right-to-Left Shunt; VAS, Visual Analog Scale.

### 3.2 Differences in ALFF

**Figure 2:**
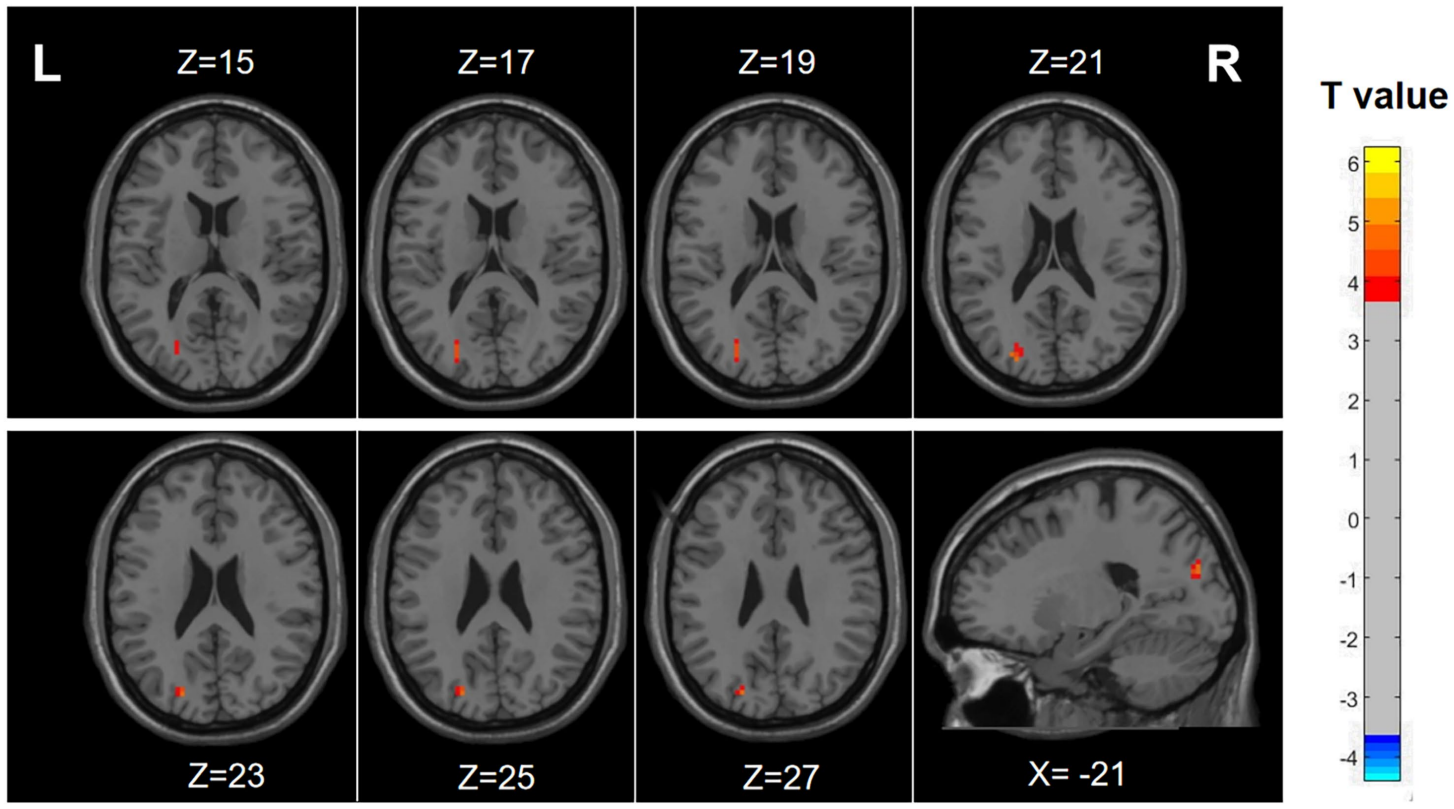
Differences in ALFF between migraine patients with and without RLS. The color red denotes increased ALFF in migraine patients with RLS compared to those without RLS. The corresponding color bar denotes the *t*-value. R, right; L, left.

The results demonstrated notably elevated ALFF in Occipital_Sup_L/Occipital_Mid_L (AAL3) regions ALFF in migraine patients with RLS in comparison to migraine patients without RLS (voxel *p* < 0.001, cluster *p*-value < 0.05, GFR corrected). Please refer to Table 2 and Figure 2.

**Table 2:** Brain regions with significant ALFF differences between migraine patients with and without RLS.

Brain regions (AAL3)	Cluster size (voxels)	Cluster volume (mm^3^)	MNI coordinates (mm)			Peak *T* value
			*X*	*Y*	*Z*	
Occipital_Sup_L Occipital_Mid_L	23	621	−21	−81	27	4.83

MNI, Montreal Neurological Institute; AAL, Anatomical Automatic Labeling.

Nevertheless, no statistically significant disparities were observed between the two groups when analyzing the fALFF and ReHo metrics.

### 3.3 Correlation of ALFF with RLS degree

**Figure 3:**
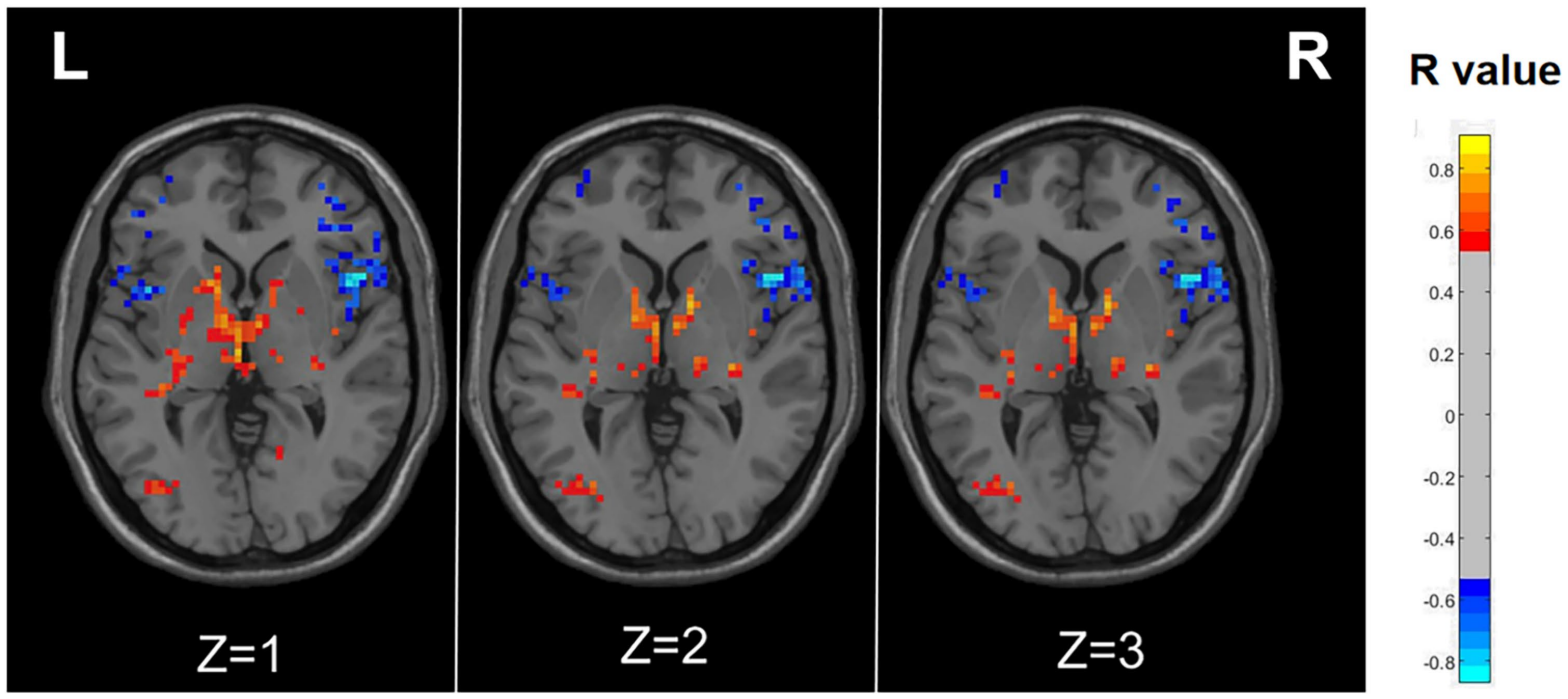
Correlation maps illustrating the relationship between ALFF and RLS degree in migraine patients with RLS. Warm colors, such as red, denote a positive correlation, while cool colors, like blue, signify a negative correlation. The color bar denotes the R-value. R, right; L, left.

As illustrated in Figure 3 and detailed Table 3, specific functional brain regions exhibited notable correlations with RLS classification. The bilateral temporal lobes (AAL3: Hippocampus_R, ParaHippocampal_R, Hippocampus_L, Temporal_Inf_L, Temporal_Sup_L), bilateral thalamus (AAL3: Thal_VL_R, Thal_VPL_R, Thal_VA_R, Thal_VL_L, Thal_MDm_L, Thal_IL_L) and left occipital lobe (AAL3: Occipital_Mid_L, Occipital_Inf_L) displayed a significant positive correlation between ALFF and RLS classification (*p* < 0.05, number of clusters >60). In contrast, ALFF in regions of right inferior frontal gyrus (AAL3: Frontal_Inf_Oper_R, Frontal_Inf_Tri_R, Frontal_Inf_Orb_2_R), bilateral middle frontal gyrus (AAL3: frontal_Mid_2_R, frontal_Mid_2_L) and bilateral insula (AAL3: Insula_L, Insula_R) exhibited a significant negative correlation with RLS classification (*p* < 0.05, number of clusters > 60).

**Table 3:** Correlation coefficients representing the relationship between ALFF and RLS degree in migraine patients with RLS.

Predominant brain areas	Cluster voxels	Peak R value
Left occipital lobe	70	0.84
Left thalamus	88	0.858
Right thalamus	66	0.91
Left temporal lobe	105	0.84
Right temporal lobe	125	0.80
Right inferior frontal gyrus	157	−0.8707
Right middle frontal gyrus	123	−0.8043
Left middle frontal gyrus	74	−0.62
Left insula	61	−0.81
Right insula	123	−0.78

**Figure 4:**
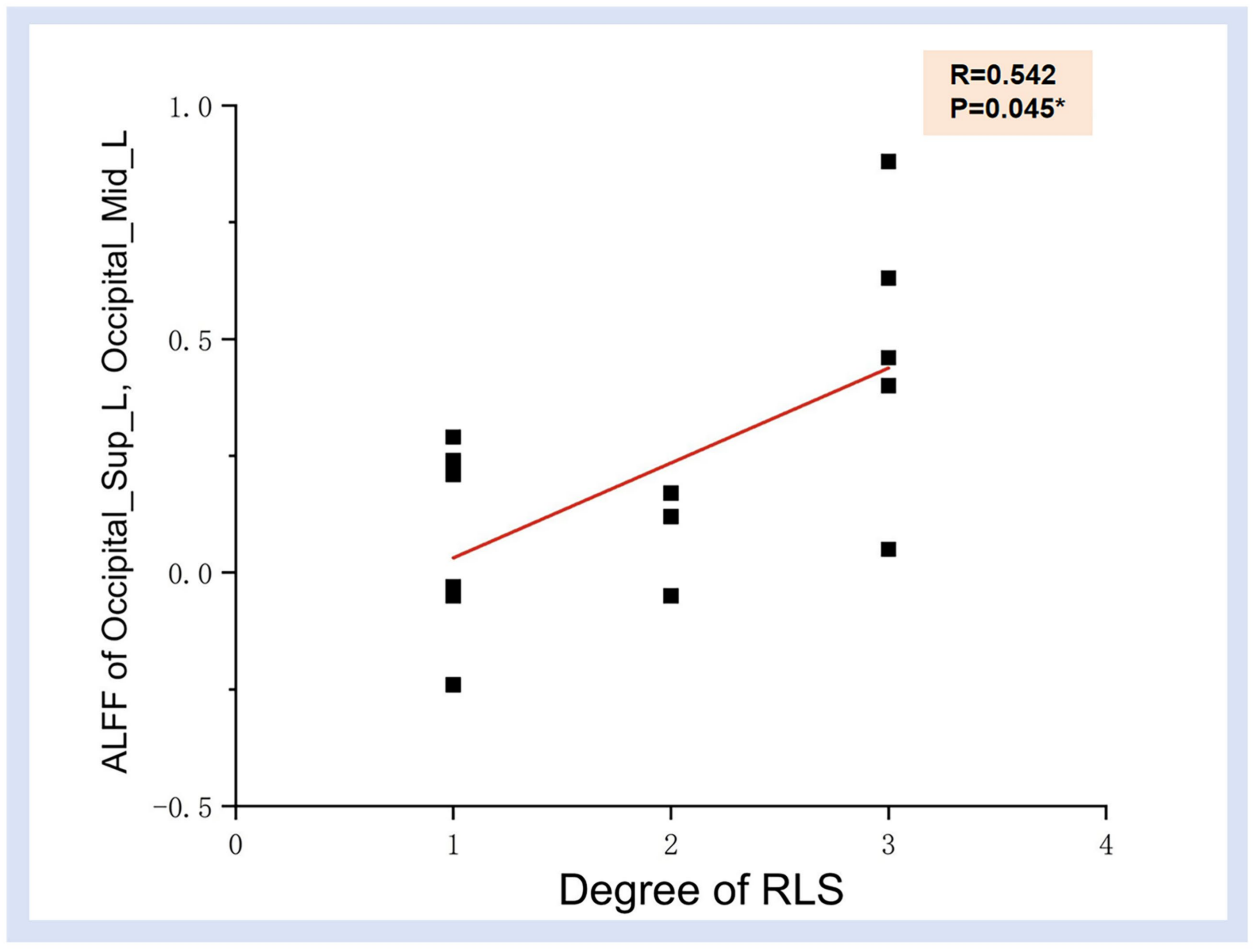
Correlation illustration between ALFF values in Occipital_Sup_L, Occipital_Mid_L regions and RLS degree. Each black dot denotes a sample. *p < 0.05. ALFF, Amplitude of Low-frequency Fluctuation; RLS, Right to Left Shunt.

The preceding findings highlight the significance of ALFF differences between the two groups, with the most prominent distinctions observed in Occipital_Sup_L and Occipital_Mid_L (AAL3). Furthermore, the ALFF values in Occipital_Mid_L and Occipital_Inf_L (ALL3) exhibited a positive correlation with RLS classification. This may imply a substantial impact of RLS on the occipital lobe. To further corroborate this observation, we extracted the ALFF values from these distinct brain regions (Occipital_Sup_L and Occipital_Mid_L) in migraine patients with RLS and assessed their correlation with RLS grading. The results, illustrated in Figure 4, indicated a positive correlation between the ALFF values of the Occipital_Sup_L and Occipital_Mid_L regions and RLS grading, with an R-value of 0.542 (*p* < 0.05).

### 3.4 Functional connectivity

**Figure 5:**
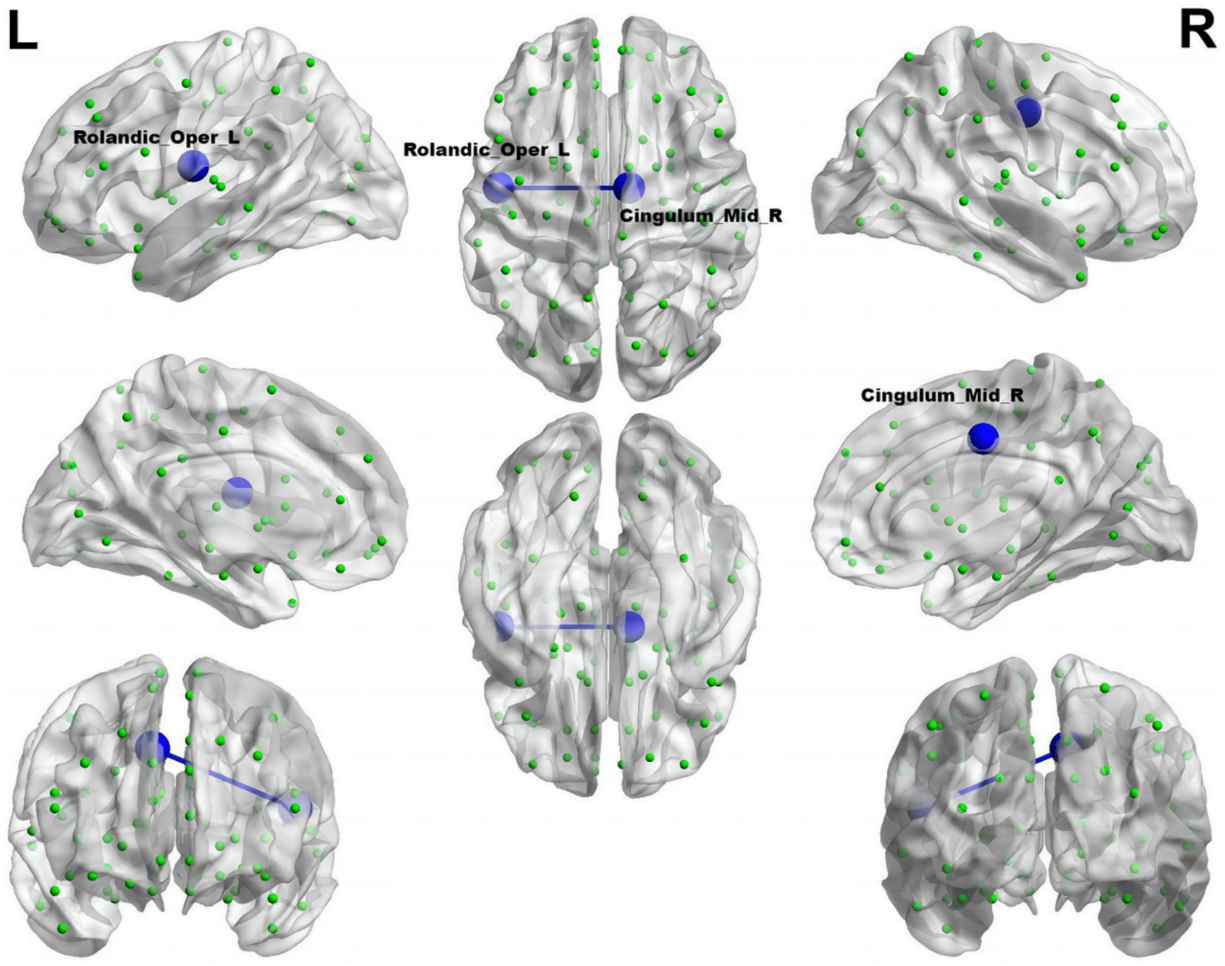
Reduced connectivity between Roland_Opper_L and Cingulum_Mid_R of the migraine group with RLS in comparison to the migraine group without RLS. The azure sphere symbolizes a node corresponding to a specific brain region, while the blue line signifies a decline in functional connectivity. R, right; L, left.

Illustrated in Figure 5, the migraine group with RLS exhibited a noteworthy reduction in functional connectivity within Roland_Opper_L and Cingulum_Mid_R (AAL), when compared to the migraine group without RLS (*p* < 0.001).

As depicted in Supplementary Figure S1, upon analyzing functional networks grounded in the Doschenbach’s 160 atlas, we discovered that the migraine with RLS demonstrated notably decreased functional connectivity in comparison to the migraine without RLS (*p* < 0.001), Specifically, this decrease was observed between key brain regions, including the ventromedial prefrontal cortex (vmPFC) within the default mode network (DMN) and the post-occipital area within the visual network (VN). Additionally, the superior temporal gyrus (also part of the DMN) exhibited reduced connectivity with the middle occipital region (visual network) and the ventral frontal cortex, belonging to the ventral attention network (VAN). Lastly, the parietal region (the somatomotor network) demonstrated decreased functional connectivity with the basal ganglia (limbic network) and temporal regions, which is affiliated with the somatomotor network (SMN). Conversely, there was a marked enhancement in the connectivity between the ventromedial prefrontal cortex (vPFC), part of the frontoparietal network (FPN), and the frontal regions, which constitute the VAN.

### 3.5 Small-world property

**Figure 6:**
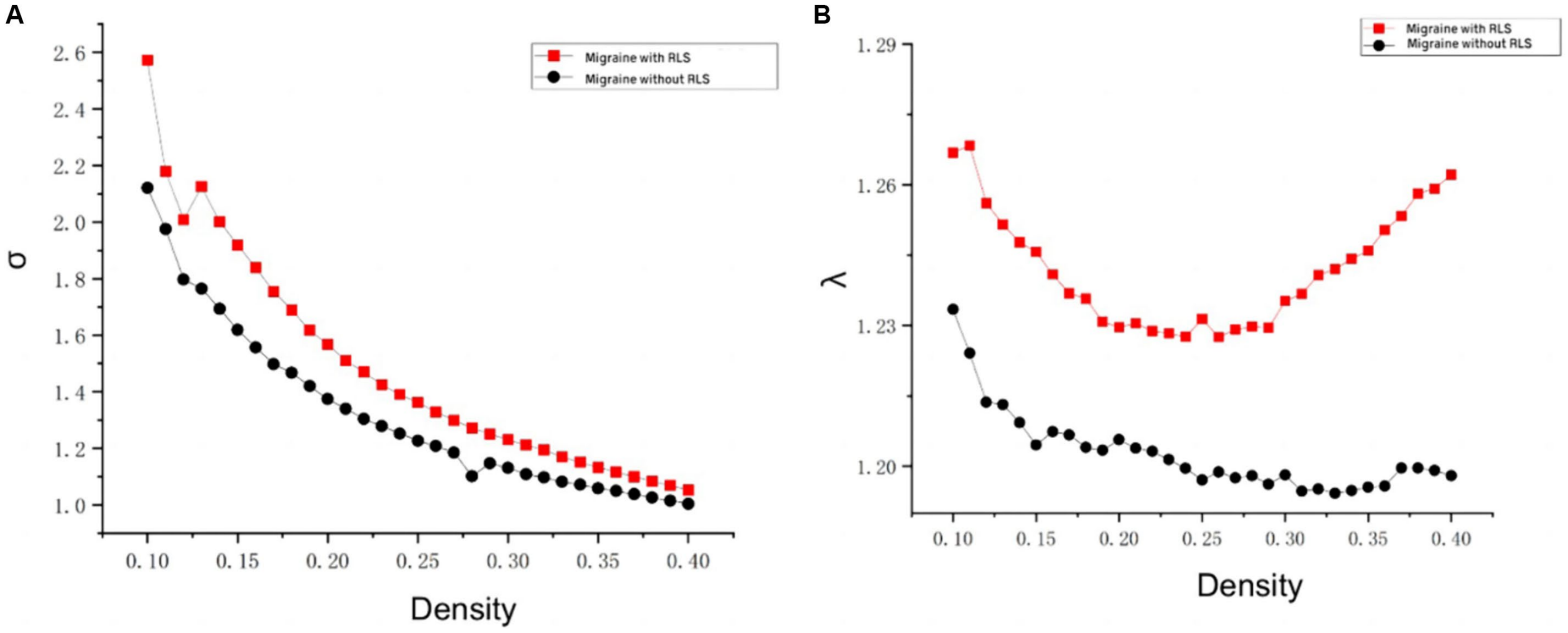
Comparison of “Small World” properties between two groups. **(A)** A comparison of “σ” property between two groups. **(B)** A comparison of “λ” property between two groups. The typical small-world network architectures, characterized by λ ≈ 1 and σ > 1, were observed across different sparsity levels. Black lines represent the migraine group without RLS, while red lines represent the migraine group with RLS. No statistically significant differences were identified between the two groups.

**Figure 7:**
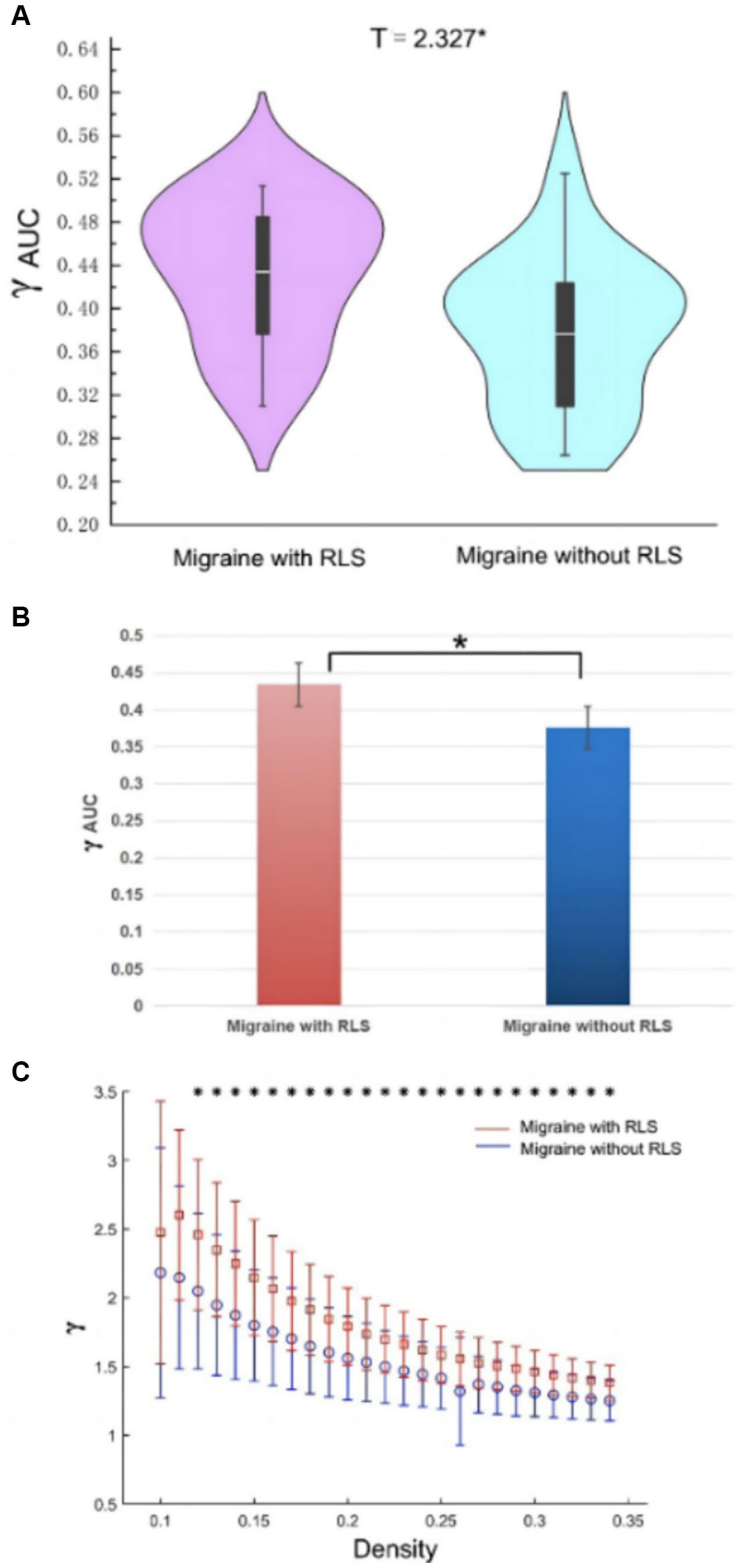
Differences in network topological properties between migraine patients with and without RLS. **(A)** Violin plots depict the distribution of mean γ AUC values, highlighting the contrast between migraine with RLS and without RLS. **(B)** The bar graph displays the significant AUC values of γ between the two groups. **(C)** γ values are shown across a density range spanning from 10 to 34%. Each point, accompanied by an error bar, represents the mean and standard deviation at specific density levels, respectively. * denotes significant differences.

The analyses of small-world properties revealed that both sets of data displayed σ greater than 1 and exhibited Cp values (λ close to 1) similar to the randomized network, as shown in Figure 6. Consequently, both groups exhibited small-world properties in their functional brain networks. Furthermore, the AUC of γ in migraine patients with RLS group was significantly higher than that of the group without RLS (*p* < 0.05), as demonstrated in Figure 7. However, the remaining indices, including σ AUC, λ AUC, E_glob_, E_loc_, C_p_, and L_p_, did not exhibit significant difference between the two groups.

Furthermore, by employing the graph theory analysis method grounded in the Doschenbach’s 160 atlas, we computed the small-world properties. However, upon comparing the two groups, our analysis failed to uncover any statistically significant differences (*p* > 0.05) in the values of γ, σ, λ E_glob_, E_loc_, C_p_, and L_p_.

### 3.6 Nodal properties

**Figure 8:**
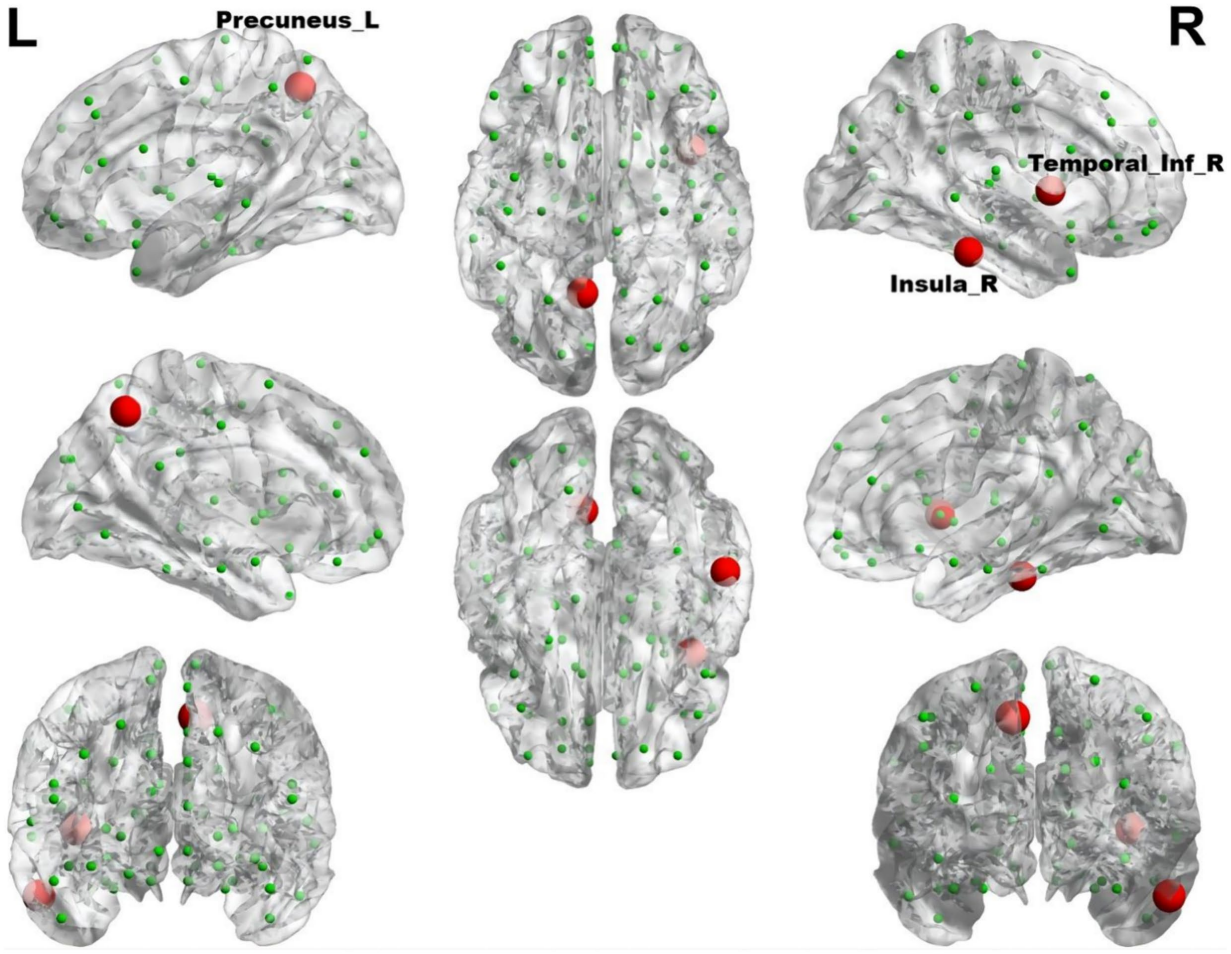
The brain regions with statistically significant difference in betweenness centrality between the migraine groups with RLS and without RLS. The red spheres represent the brain nodes with increased betweenness centrality in migraine with RLS compared to migraine without RLS. R, right; L, left.

In comparison to control subjects, the migraine group with RLS exhibited significantly elevated nodal Betweenness centrality in the Insula_R, Precuneus_L, and Temporal_Inf_R (ALL) (*p* < 0.01), as demonstrated in Figure 8. However, no significant disparities were found in nodal degree and nodal efficiency between the two groups.

**Figure 9:**
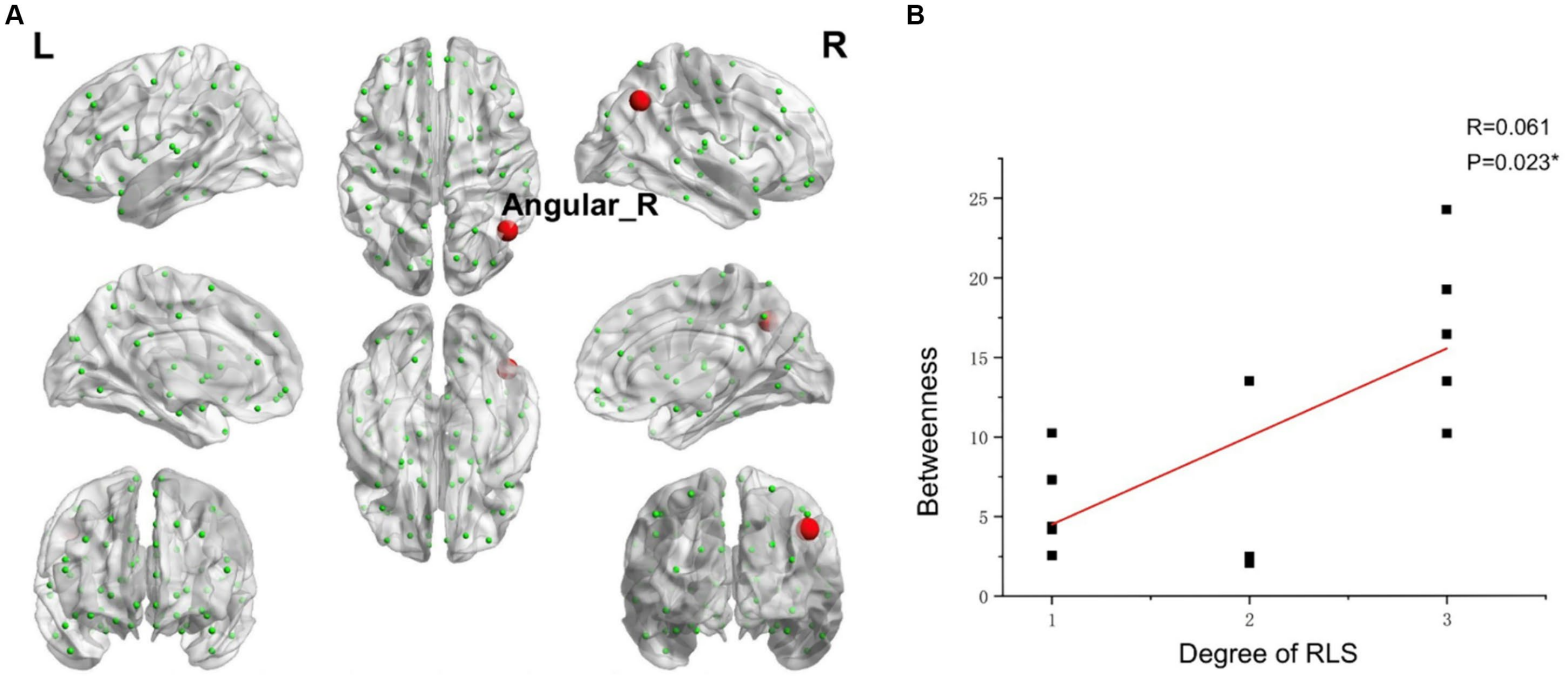
Correlations between the betweenness centrality and RLS grading. **(A)** Brain map depicting node with positive correlation. **(B)** Correlation graph. *p < 0.05. RLS, Right-to Left Shunt. Red node represents those demonstrating a correlation between betweenness centrality and RLS grading. Each black dot signifies an individual sample.

Using RLS grading as the variable, and accounting for age and head movement coefficients as covariates, we employed the DPABI NET toolbox to investigate potential correlations between the brain topological metrics and RLS grading within the migraine patients with RLS group (*p* < 0.05). The results demonstrated that RLS grading is positively correlated with the betweenness centrality of Angular_R (AAL) (*p* < 0.01) (Figure 9). However, none of the seven topologic small-world parameters including σ AUC, Ƴ AUC, λ AUC, L_p_ AUC, C_p_ AUC, E_glob_ AUC, and E_loc_ AUC exhibited a significant correlation with RLS grading. Furthermore, no noteworthy correlation was observed between nodal degree and RLS grading.

Using the Doschenbach’s 160 atlas, we discovered a notably (*p* < 0.01) lower nodal degree in the occipital lobe among migraine patients with RLS, as compared to those without RLS (Supplementary Figure S2). Additionally, migraine patients with RLS demonstrated a marked decrease (*p* < 0.01) in nodal efficiency within the fusiform gyrus, in contrast to migraine patients without RLS (Supplementary Figure S3).

## 4 Discussion

Our primary objective is to assess RLS-related functional alterations at both localized and global levels in individuals with migraines and RLS. We conduct an analysis to discern the disparities in brain functional segregation among migraine patients with and without RLS, utilizing indicators such as ALFF, fALFF, and ReHo. Concurrently, we appraise the variations in brain network integration function by employing structural templates like AAL and functional templates such as Doschenbach’s 160 atlas. The following chapters will meticulously explore the intricacies of these functional alterations and delve into the correlations between these brain functional changes and both RLS and migraine.

### 4.1 ALFF difference between two groups

Our findings indicate a significant increase in ALFF, particularly in the left middle occipital gyrus and superior occipital gyrus, within the RLS-positive group, with a positive correlation observed with RLS severity. Despite the absence of statistically significant disparities between the two groups concerning fALFF and ReHo metrics, we posit that the observed results could potentially be attributed to the constrained sample size. ALFF, reflecting the intrinsic neural activity of the brain, serves as a valuable tool for assessing the functional attributes of specific disease-associated brain regions (Zang et al., 2007; Liu K. et al., 2020; Liu L. et al., 2020; Wang et al., 2023; Zhe et al., 2023). Elevated ALFF values signify heightened spontaneous neural activity in the brain area, while reduced ALFF values indicate decreased spontaneous activity. A recent investigation reported an increased detection rate of RLS in individuals experiencing migraines with visual aura, underscoring a robust correlation between RLS and visual aura (Khessali et al., 2012). Furthermore, Tu et al. previously identified substantial functional connectivity anomalies involving the occipital cortex, precuneus, and thalamus in migraine patients through dynamic analysis of brain functional connectivity (Tu et al., 2019). In alignment with our current study, we uncovered augmented spontaneous activity in the middle occipital and superior occipital gyrus among migraine patients with RLS in comparison to those without RLS, with a notable correlation observed concerning the severity of RLS. Hence, we posit that the accumulation of microemboli and vasoactive substances, engendered by RLS, within the occipital lobe may be contributing to the observed alterations in spontaneous brain activity. This phenomenon may also elucidate the heightened likelihood of visual aura manifestation in patients with RLS.

The pain transmission pathway comprises three neuronal levels: peripheral primary neurons initially detect injurious stimuli and relay them via afferent fibers to interneurons. Subsequently, this information is conveyed through the spinothalamic tract and spinoreticular tract to tertiary neurons residing in the thalamus, the central sensory hub. Ultimately, the thalamus transmits the noxious signals to various regions of the cerebral cortex, including the bilateral insulae, prefrontal cortex, anterior cingulate cortex, and somatosensory cortex (Liu et al., 2012; Baron et al., 2010). The trigeminal vascular theory, stemming from this foundational model, currently serves as the predominant framework for investigating migraine mechanisms (Lee and Neumeister, 2020). At its core, this theory hinges on a three-tiered neuronal mechanism, encompassing the trigeminal ganglia (primary neurons), brainstem neurons (intermediate neurons), and thalamus (tertiary neurons). Ultimately, the thalamus dispatches nociceptive information to various regions within the cerebral cortex, including the auditory cortex, olfactory cortex, insular cortex, primary motor cortex, secondary motor cortex, periaqueductal gray matter, posterior subcortical cortex, parietal cortex, primary somatosensory cortex, secondary somatosensory cortex, primary visual cortex, secondary visual cortex, and more. This intricate interplay generates the sensation of pain (Khan et al., 2021). In our present investigation, we observed significant correlations between RLS grading and ALFF in the thalamus, as well as the occipital, prefrontal, and insular cortex regions. Importantly, several of these associations exhibited positive correlations. These findings signify that RLS may influences the pain transmission pathways, thereby reinforcing the association between RLS and migraine.

### 4.2 Functional connectivity difference between two groups

The rostral anterior cingulate cortex assumes a pivotal role in pain modulation, serving as a linchpin in the downstream pain inhibitory system. Li and colleagues have previously reported a reduced resting-state functional connectivity between the rodent anterior cingulate cortex (rACC)/medial prefrontal cortex (mPFC) and periaqueductal gray (PAG) in migraine patients compared to healthy controls (Ashina et al., 2019). In alignment with these findings, our results also indicate decreased connectivity involving the central sulcus and the medial and paracingulate cingulate gyrus, as well as vmPFC and post occipital in RLS-positive group compared with RLS-negative group. More importantly, the cingulate gyrus primarily governs the regulation of visceral functions, potentially elucidating the common presence of nausea and vomiting symptoms alongside migraine, attributed to the abnormal visceral regulation orchestrated by the cingulate gyrus (Li et al., 2016). Furthermore, the number of brain regions exhibiting functional connectivity differences is greater when utilizing the Doschenbach’s 160 atlas compared to the AAL template. Our research delved deeper to uncover a significant decline in functional connectivity among migraine patients with RLS. This encompassed a notable reduction in the interplay between the DMN and the VN as well as the VAN, coupled with a substantial weakening of internal functional connectivity within the SMN, and its connections to the limbic network (LN). Interestingly, migraine patients with RLS exhibited a significantly enhanced functional connectivity between the FPN and the VAN, in comparison to those without RLS. Numerous studies have consistently shown pronounced abnormalities in the default mode network, somatomotor network, visual network and limbic network among individuals suffering from migraine, highlighting their crucial roles in pain perception and modulation (Messina et al., 2022; Hu et al., 2023; Zhang et al., 2016; Yu et al., 2017; Colombo et al., 2015; Xue et al., 2012). Our research extends these findings by suggesting that RLS may be intricately linked to migraine through its impact on the brain networks that regulate pain.

Thus, we speculate that RLS may have a potential impact on the pain regulatory pathway in migraine patients.

### 4.3 Difference in small-world properties and nodal properties

Graph theory analysis is increasingly applied in migraine research, with several studies elucidating the small-world properties of functional brain networks in various migraine conditions, including migraine without aura and chronic migraine (Liu et al., 2012; Li et al., 2017; Dai et al., 2021). Our investigation, too, revealed that both migraine groups, with and without RLS, exhibited small-world properties within their functional brain networks. However, substantial distinctions in topological properties remained evident between the two cohorts. Notably, the RLS-positive group displayed higher γ values, indicative of intensified small-world properties and enhanced efficiency in brain network message transmission. This heightened efficiency sustains an optimal equilibrium between functional segregation and integration. It is plausible that these differences in network topology are attributable to RLS, fostering a more streamlined information exchange within the brain network. Consequently, this refined network may perceptively and expeditiously transmit pain signals among pain-related brain regions.

Betweenness centrality, an index signifying local network attributes, gages a node’s influence on information transmission amid other nodes. The nodal degree provides an assessment of the information integration capacity of a particular brain region within the network, effectively quantifying its significance and importance (Yan et al., 2013). While we have scrutinized the overall small-world characteristics of the brain network in migraine patients with RLS, it remains imperative to delve deeper into the potential alterations within specific local nodes responsible for information transfer in the functional brain network, possibly influenced by RLS. In contrast to the RLS-negative group, our study detected heightened betweenness centrality of left precuneus, right inferior temporal gyrus and insula in the group afflicted by RLS. The insula’s pivotal role in triggering the pain matrix network is well-documented (Labrakakis, 2023). Functioning as a hub within the cortex, the insula orchestrates complex sensations and emotions associated with migraine symptoms. This is accomplished through intricate interconnections linking the frontal, temporal, and parietal cortices, basal ganglia, thalamus, and limbic structures (Borsook et al., 2016). Moreover, the inferior temporal gyrus plays a fundamental role in the assembly of the ventral visual pathway, primarily responsible for shaping visual content (Li et al., 2018). Our study revealed that RLS-positive group exhibited significantly elevated betweenness centrality in this region compared to RLS-negative group. Thus, it is plausible that RLS’s influence extends to the inferior temporal gyrus, rendering migraine patients with RLS more susceptible to visual aura. This aligns with prior research by Kijima et al., wherein they reported higher RLS grades in the migraine group with frequent visual aura compared to those with episodic visual aura or no visual aura (Kijima et al., 2015). Research demonstrates that the fusiform gyrus plays a crucial role in intricate visual processing tasks, encompassing color perception and shape recognition among others (Schankin et al., 2020). Interestingly, our study uncovered a marked decrease in the nodal degree within the occipital lobe and a substantial decline in the nodal efficiency of the fusiform gyrus among migraine patients with RLS. These discoveries imply a compromised capacity of the occipital lobe to integrate information within the intricate brain network, coupled with a diminished efficiency in the fusiform gyrus’s elaboration of visual stimuli. Consequently, we posit that RLS might adversely affect the visual processing prowess and efficiency of both the occipital lobe and the fusiform gyrus, ultimately giving rise to visual abnormalities. Consequently, our study further supports the association between RLS and migraine, viewed through the lens of brain function.

The angular gyrus, positioned within the parietal lobe, emerges as a pivotal structure implicated in the intricate process of multisensory integration. It serves as a central hub orchestrating the harmonious synthesis of information from diverse sensory modalities across the cerebral landscape (Dong et al., 2023; de Pasquale et al., 2012). Our comprehensive data analysis, notably, illuminated a conspicuous and statistically significant correlation between the betweenness centrality of the angular gyrus and the grading of RLS. This observation implies a possible association between RLS and sensory integration in migraine.

### 4.4 Limitations and future work

This study warrants acknowledgment of certain potential limitations. To commence, the relatively modest sample sizes within both study groups may introduce constraints on the precision and generalizability of the findings. Secondly, the lack of a control group without migraine but with and without a RLS prevents us from detecting subtler changes related to RLS in migraine patients. Future research endeavors should consider broader subject recruitment, encompassing a more extensive cohort of participants, particularly the inclusion of healthy controls, as well as the utilization of multimodal neuroimaging analysis, which encompass both structural MRI, diffusion tensor imaging (DTI) and task-state fMRI, to enhance the comprehensiveness and depth of the investigations. We should further incorporate healthy control with and without RLS, to explore whether there is an effect of RLS on brain function, which may induce migraine attacks. This will help us to verify whether RLS is a risk factor for migraine. Thirdly, because of the absence of the information of heartbeat and respiration during rs-fMRI scans, the potential confounding effect of heartbeat and respiration were not regressed. Heartbeat and respiratory signals should be collected in future research to make the results more rigorous. Fourthly, it is essential to recognize that this study adhered to a cross-sectional design. Consequently, it cannot elucidate the causal dynamics underpinning the observed aberrations in brain functional imaging. In other words, it remains uncertain whether these anomalies precede the onset of migraine or are sequelae triggered by recurrent migraine episodes.

## 5 Conclusion

In conclusion, this study elucidated RLS-associated brain functional changes, including ALFF, FC and topological properties in individuals with migraine and RLS. From the perspective of functional segregation and integration, the migraine with RLS exhibited significantly different local spontaneous neural activity, integration across distinct brain regions and functional networks features compared to migraine without RLS. Furthermore, migraine with RLS showed a notable correlation between RLS grading and ALFF in certain brain regions. Additionally, RLS severity is positively correlated with the angular gyrus betweenness centrality. This study enhances our understanding of RLS-related migraine and introduces a novel method for exploring the potential brain functional alterations in migraine with RLS.

## Data Availability

The raw data supporting the conclusions of this article will be made available by the authors, without undue reservation.

## Footnotes

^1^ http://rfmri.org/dpabi

^2^ https://www.nitrc.org/projects/bct/

^3^ https://www.nitrc.org/projects/bnv/

^4^ https://www.ibm.com/spss
