# Supplementary material for: Right-to-left shunt-associated brain functional changes in migraine: evidences from a resting-state FMRI study
Source: Front Hum Neurosci. 2024 Aug 30;18:1432525. doi: 10.3389/fnhum.2024.1432525 (PMC11392749; doi:10.3389/fnhum.2024.1432525)
Supplement: Supplementary file 1 [file Data_Sheet_1.PDF]

Supplementary Material for ’ Right-to-Left Shunt-associated Brain Functional Changes in Migraine: Evidences from a Resting-state FMRI Study’

Wenfei Cao[1], Lei Jiao[1], Huizhong Zhou[1], Jiaqi Zhong[1], Nizhuan Wang[2, 3*], Jiajun Yang[1*]

1 Department of Neurology, Shanghai Sixth People's Hospital Affiliated to Shanghai Jiao Tong University School of Medicine, Shanghai, China
2 Department of Chinese and Bilingual Studies, The Hong Kong Polytechnic University, Hong Kong, China
3 School of Biomedical Engineering, ShanghaiTech University, Shanghai, China

1 Supplementary Figures

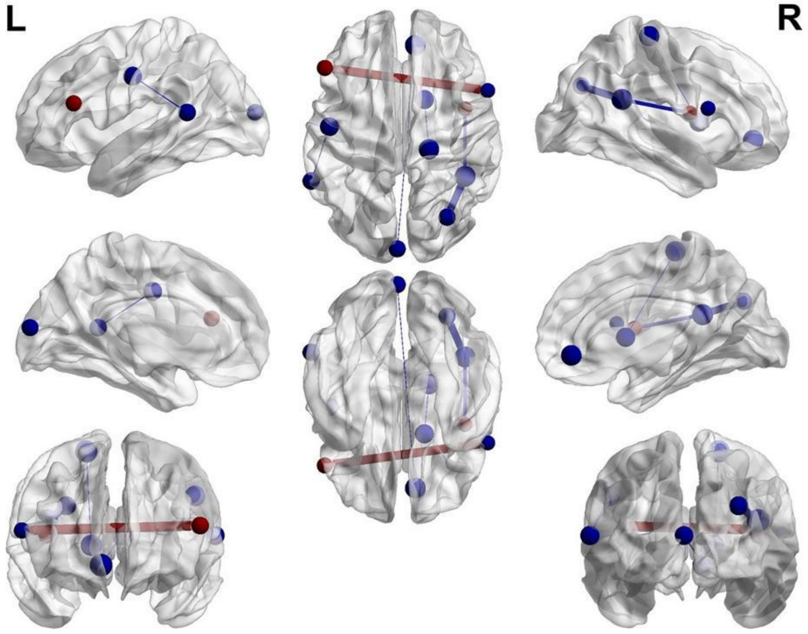

Figure S1. Intergroup differences in functional connectivity based on the Doschenbach’s 160 atlas between subjects with and without RLS. The azure sphere symbolizes a node corresponding to a

specific brain region, while the blue line signifies a decline in functional connectivity, the red line signifies an enhancement in functional connectivity. R: right; L: left.

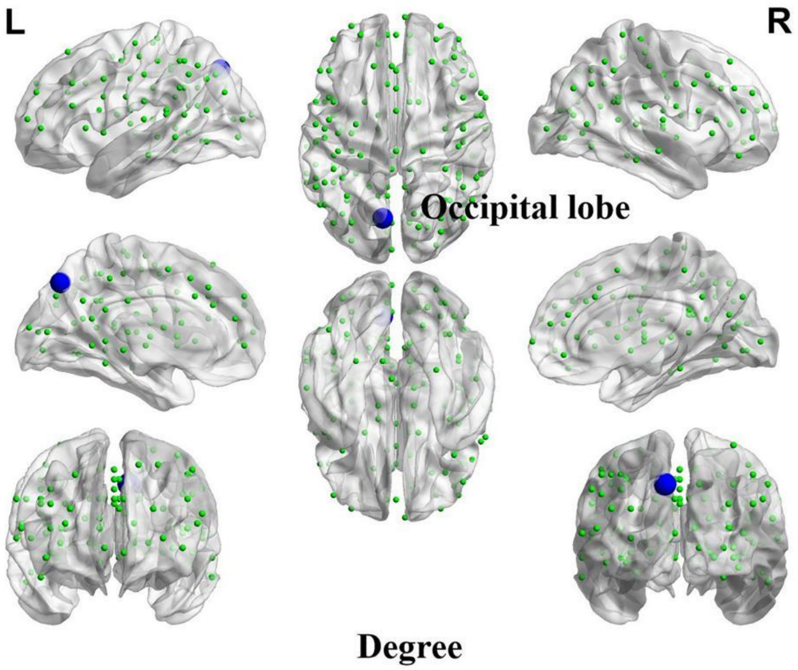

Figure S2. Group difference in nodal degree based on the Doschenbach's 160 atlas. The blue spheres represent the brain nodes with decreased nodal degree in migraine with RLS compared to migraine without RLS. R: right; L: left.

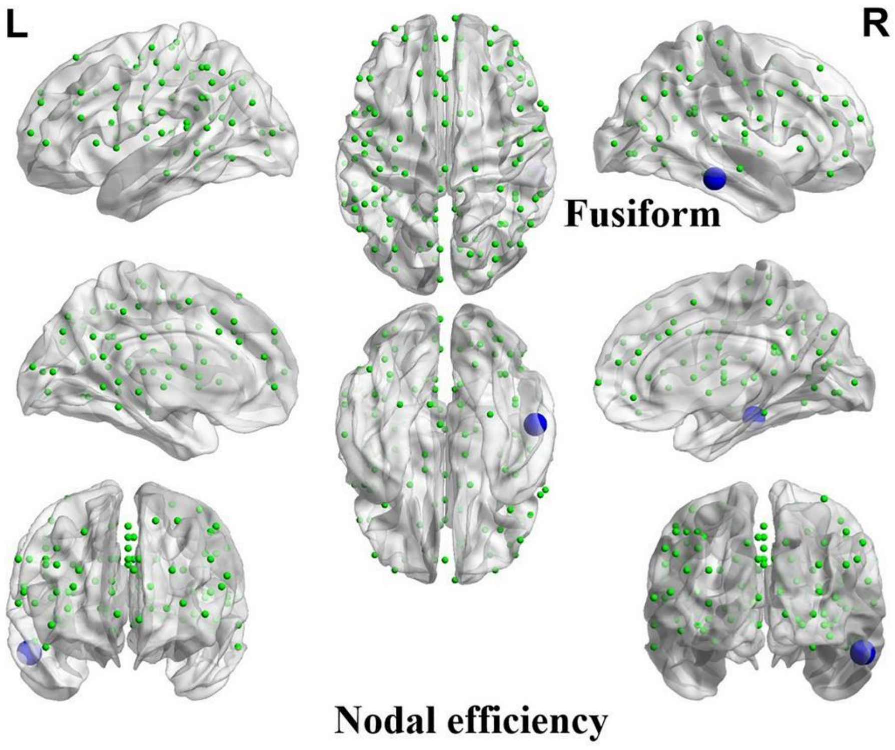

Figure S3. Group difference in nodal efficiency based on the Doschenbach's 160 atlas. The blue spheres represent the brain nodes with decreased nodal efficiency in migraine with RLS compared to migraine without RLS. R: right; L: left.
